# MobC of conjugative RA3 plasmid from IncU group autoregulates the expression of bicistronic *mobC-nic* operon and stimulates conjugative transfer

**DOI:** 10.1186/s12866-014-0235-1

**Published:** 2014-09-04

**Authors:** Jolanta Godziszewska, Anna Kulińska, Grażyna Jagura-Burdzy

**Affiliations:** Institute of Biochemistry and Biophysics, Department of Microbial Biochemistry, PAS, 02-106 Warsaw, Poland; Present address: Warsaw University of Technology, Faculty of Chemistry, Warsaw, Poland

**Keywords:** Broad-host range plasmid, Conjugative transfer, IncU, MobC, Transfer auxiliary protein

## Abstract

**Background:**

The IncU conjugative transfer module represents highly efficient promiscuous system widespread among conjugative plasmids of different incompatibility groups. Despite its frequent occurrence the mechanisms of relaxosome formation/action are far from understood. Here we analyzed the putative transfer auxiliary protein MobC of the conjugative plasmid RA3 from the IncU incompatibility group.

**Results:**

MobC is a protein of 176 amino acids encoded in the bicistronic operon *mobC-nic* adjacent to *oriT*. MobC is homologous to prokaryotic transcription factors of the ribbon-helix-helix (RHH) superfamily. Conserved LxxugxNlNQiaxxLn motif clusters MobC with the clade of conjugative transfer auxilliary proteins of Mob_P_ relaxases. MobC forms dimers in solution and autoregulates the expression of *mobCp* by binding to an imperfect palindromic sequence (O_M_) located between putative -35 and -10 motifs of the promoter. Medium-copy number test plasmid containing the *oriT-mobCp* region is mobilized with a high frequency by the RA3 conjugative system. The mutations introduced into O_M_ that abolished MobC binding *in vitro* decreased 2-3 fold the frequency of mobilization of the test plasmids. The deletion of O_M_ within the RA3 conjugative module had no effect on transfer if the *mobC-nic* operon was expressed from the heterologous promoter. If only *nic* was expressed from the heterologous promoter (no *mobC*) the conjugative transfer frequency of such plasmid was 1000-fold lower.

**Conclusion:**

The MobC is an auxiliary transfer protein of dual function. It autoregulates the expression of *mobC-nic* operon while its presence significantly stimulates transfer efficiency.

**Electronic supplementary material:**

The online version of this article (doi:10.1186/s12866-014-0235-1) contains supplementary material, which is available to authorized users.

## Background

Broad-host-range conjugative plasmids are considered the main factors responsible for the horizontal spreading of genetic information between distantly related bacterial species. Although the conjugation process has been fairly well described for some model systems like F, Ti, R388 or RK2 [1–5], its regulation and the environmental stimuli responsible for the initiation of conjugation remain elusive. In Gram-negative bacteria the conjugation functions comprise processing of DNA for transfer (Dtr) and mating pair formation (Mpf). Among Dtr proteins the pivotal role is played by relaxase which recognizes a specific motif in *oriT* (origin of transfer), nicks a single DNA strand, covalently binds to the 5′ end of the transferred strand (T-DNA) and re-joins the ends after ssDNA translocation to the recipient. Proteins involved in Dtr and Mpf functions form two large complexes: relaxosome (relaxase bound at specific DNA sequence *oriT* and auxiliary proteins) [5] and membrane located transferosome (type IV secretion system, T4SS) [6]. The third essential element of conjugative transfer system is a coupling protein (T4CP) that links the relaxosome with the transferosome [7]. The auxiliary proteins help to determine the specificity of *oriT* recognition by relaxase, enhance its nicking activity, stimulate ATPase activity of the coupling protein and act as transcriptional regulators for conjugative transfer operons [8–19].

**Figure 1:**
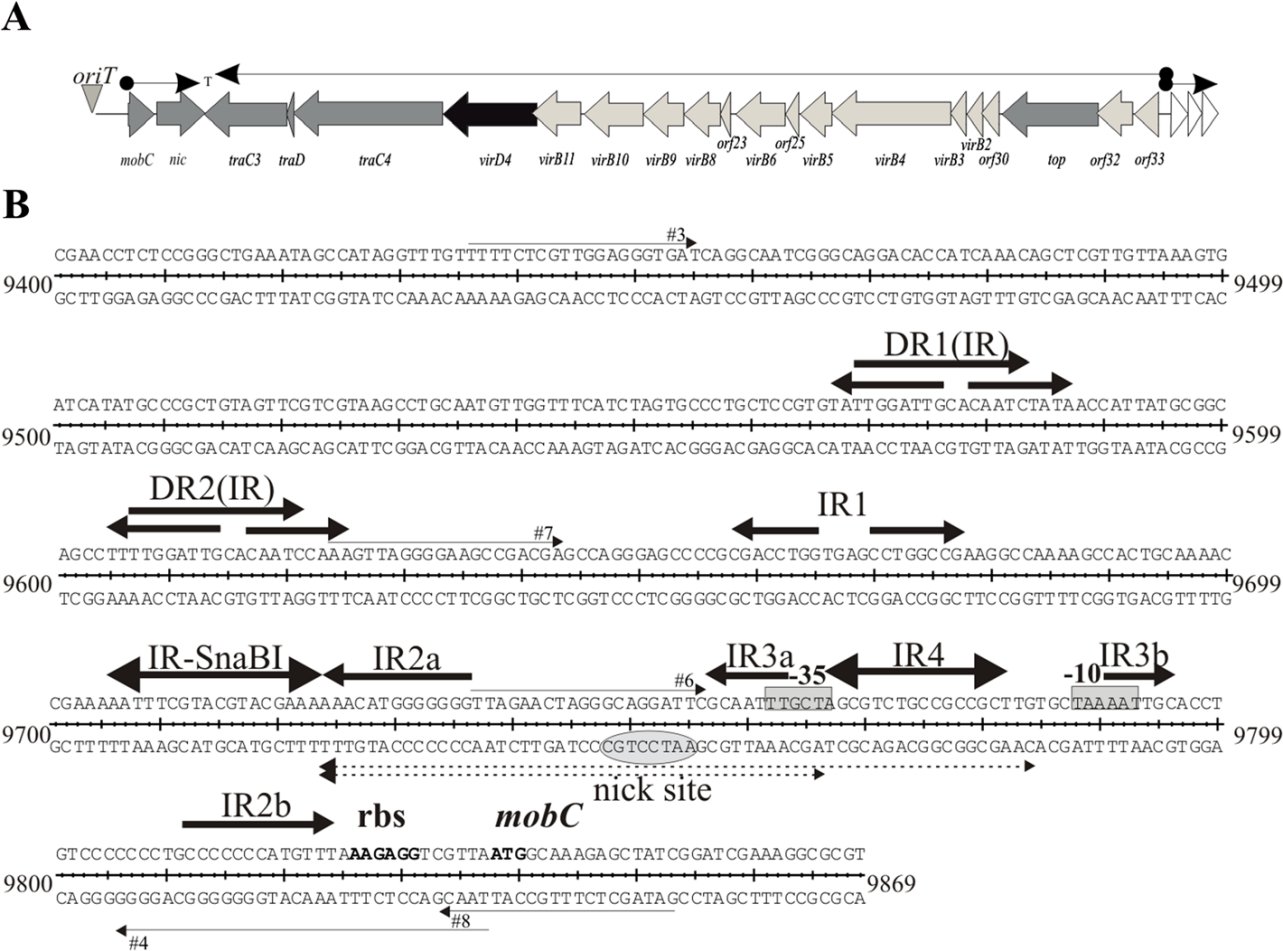
**Organization of the RA3 conjugative transfer module. A**. Transcriptional organization of the RA3 conjugative transfer module with orfs labeled according to the predicted function in the conjugation process: light grey arrows indicate homologs of Mpf system, dark grey arrows indicate proteins presumably involved in DNA replication and transfer (Dtr), and the black one marks a homolog of coupling protein VirD4. **B**. DNA sequence of *mobCp/oriT/parS* region between 9400 and 9869 nt of RA3 coordinates [GenBank: DQ401103]. Thin arrows correspond to primers used in the construction of deletion derivatives by PCR. Putative promoter motifs are boxed, and putative regulatory sequences are indicated by arrows. The inverted repeat IR-SnaBI is a part of the centromere-like sequence as the binding site for partitioning protein KorB (O_B_) [43]. Highly conserved nick site is circled and the ribosome binding site and initiation codon for MobC are in bold. The oligonucleotides tested as *oriT*s (61 nt and 45 nt) are shown.

The broad-host-range conjugative plasmid RA3, the archetype of the IncU incompatibility group, has been isolated from the fish pathogen *Aeromonas hydrophila* as the determinant of its antibiotic resistance [20]. RA3 nucleotide sequence has been determined [GenBank: DQ401103]; [21] and shown to be almost identical in its backbone part to another IncU representative pFBAOT6 [22]. The RA3 plasmid has been shown to be capable of replicating and self-transmitting between α-, β- and γ- proteobacteria with a very high efficiency [21]. The conjugative transfer functions of RA3 are clustered in a region of 23 kb organized in three transcriptional units (Figure 1A). The first operon encodes two DNA- transfer associated products (Dtr): a predicted DNA- binding protein MobC (176 amino acids) and the relaxase Nic (331 amino acids). The sequence corresponding to the putative nick site of *oriT* has been identified *in silico* upstream of the *mobC-nic* operon [21]. It was then confirmed that when an intergenic fragment of 417 bp containing putative *oriT*_RA3_ (Figure 1B) was cloned into pUC18 [23], it facilitated plasmid mobilization by the RA3 with a high frequency [21].

The second transfer operon located on the opposite DNA strand encompasses 19 open reading frames encoding proteins mainly involved in the mating pair formation (Mpf), four putatively in Dtr functions (homologs of RP4 primases TraC3 and TraC4, TraD and DNA topoisomerase Top), and a homolog of the coupling protein VirD4 [21]. The third operon contains three orfs *orf34, orf35* and *orf36* (Figure 1A) encoding a predicted membrane associated proteins and a DNA binding protein [21], that fulfill the auxilliary transfer functions (J. Godziszewska, unpublished).

**Figure 2:**
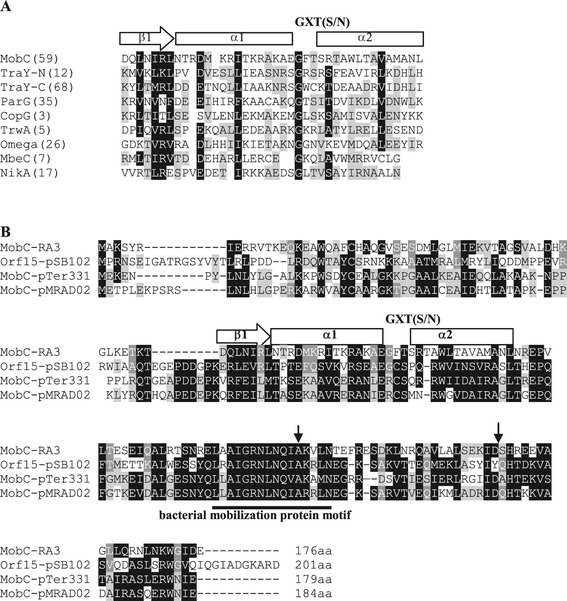
**Amino acid sequence of MobC protein from RA3 (IncU). A**. Ribbon-helix-helix (RHH) motifs of the plasmid proteins involved in conjugative transfer or stable maintenance: TraY of F [Uniprot:P06627]; ParG of TP228 [Uniprot: Q70W75], CopG of *Streptococcus* plasmid pLS1 [Uniprot:P13920], TrwA of R388 [Uniprot: Q04229], Omega of pSM19035 [Uniprot: Q83UM6], MbeC of ColE1 [Uniprot: P13657] and NikA of R64 [Uniprot: Q79VV8]. The numbers in brackets correspond to the first amino acid residue of RHH motif. Similar residues in seven to nine representatives are shadowed in white on black, those similar in four to six representatives are shadowed grey. **B**. MobC family of putative conjugative auxiliary proteins from the IncU plasmids (due to the high similarity only MobC of RA3 is shown as the representative of IncU group) [GenBank: ABD64841] and PromA groups (Orf15 of pSB102 [GenBank: NP_361029], MobC of pTer331 [GenBank: YP_001672038] and MobC of pMRAD02 [GenBank: ACB28263]. Putative RHH motif and a highly conserved “mobilization protein motif” are shown. Similar residues in three or four representatives are shadowed in white on black, those similar in two are shadowed grey. Black arrows mark the C-termini of truncated MobCs analyzed in this work.

We initiated a functional analysis of the RA3 conjugative module to uncover the roles of individual elements in this extremely efficient transfer system [21] starting from the operon *mobC-nic*. The closest homologs of MobC from IncU plasmids [22,24] have been found encoded in the similarly organized transfer regions of promiscuous plasmids from a putative new plasmid group, designated PromA [25]. They all have been intuitively classified as the auxiliary transfer protein (Figure 2B) [25].

The best characterized auxiliary transfer proteins are TraJ, TraK and TraH of RK2 [26], TraY and TraM of F [8,10,27], TrwA of R388 [28] and MbeC of ColE1 [13]. They ensure specificity of the relaxase binding to the nick site, change the topology of DNA by bending, enhance the nicking reaction, stimulate unwinding of *oriT*, and stabilize the relaxosome [8–12]. It has been shown that C-terminal domain of TrwA stimulates ATPase activity of TrwB, the coupling protein of R388 conjugative system [14]. The role of the auxiliary proteins in the relaxosome formation/activity is essential since deletions of the coding regions or their binding sites decreased up to 10^5^ the frequency of plasmid mobilization [19,29,30]. In many cases the auxiliary transfer proteins also act as transcription factors repressing or activating the expression of *tra* genes [15–17,19].

In this work we have analyzed the role of MobC in the conjugative transfer of RA3 plasmid showing that it is an auxiliary transfer protein of dual function. MobC controls the expression of *mobC-nic* operon by binding to the operator sequence in the *mobCp* and is required for the fully efficient conjugation process.

## Methods

### Bacterial strains and growth conditions

*Escherichia coli* strains used were DH5α [F^*-*^(*Φ80*d*lacZΔM15*) *recA1 endA1 gyrA96 thi-1 hsdR17*(*r*_*k*_^*-*^*m*_*k*_^*+*^) *supE44 relA1 deoR Δ(lacZYA-argF)U196*] and its Rif^R^ derivative, BL21 [F^-^*ompT hsdS*_B_ (r_B_^-^m_B_^-^) *gal dcm* (λ DE3)] (Novagen, 2003); and BTH101 [F^*-*^, *cya-99 araD139 galE15 galK16 rpsL1* (Sm^R^) *hsdR2 mcrA1 mcrB1*] [31]. Bacteria were grown in L-broth [32] at 37°C or on L-agar (L-broth with 1.5% w/v agar) supplemented with appropriate antibiotics: benzyl penicillin, sodium salt (150 μg ml^-1^ in liquid media and 300 μg ml^-1^ in agar plates) for penicillin resistance, kanamycin 50 μg ml^-1^ for kanamycin resistance and chloramphenicol 10 μg ml^-1^ for chloramphenicol resistance. MacConkey Agar Base (Difco) supplemented with 1% maltose was used for bacterial adenylate cyclase two-hybrid (BACTH) system. L agar used for blue/white screening contained IPTG (0.1 mM) and X-gal (40 μg ml^-1^).

### Plasmid DNA isolation, analysis, cloning and manipulation

Plasmid DNA was isolated and manipulated by standard procedures [33]. Plasmids used and constructed in this study are listed in Table 1. Standard PCR reactions [34] were performed with pairs of primers listed in the Additional file 1. PCR reactions to amplify *mobC* were performed with an initial denaturation step (95°C for 5 minutes) and 25 cycles of denaturation at 95°C for 30 seconds, annealing at 55°C for 30 seconds and elongation at 72°C for 40 seconds; for amplification of *mobCp* fragment and its derivatives annealing was performed at 59°C for 30 seconds. Reactions ended with a final elongation step (72°C for 7 minutes). All PCR- derived clones were sequenced to verify the nucleotide sequence.

**Table 1:** **Plasmids used in this study**

**Designation**	**Relevant features**	**Copy number**	**References**
pBBR1MCS-1	BHR^1^, IncA/C*,* Cm^R^	Medium	[36]
pBGS18	*ori*_MB1_, Km^R^	High	[41]
pET28a	*ori*_MB1_, Km^R^, T*7p*, *lacO*, His_6_- tag, T7 tag	Medium	Novagen
pGBT30	*ori*_MB1_, Ap^R^, *lacI* ^q^, *tacp* expression vector	High	[35]
pJSB1.24	*ori*_MB1_, Km^R^, Tra_RA3_-*korCp-korC* (RA3 coordinates 9437-33657 nt, 3093-3705 nt)	High	[42]
pKGB4	pUT18 with MCS modified	High	Głąbski K.^2^
pKGB5	pKNT25 with MCS modified	Medium	Głąbski K.^2^
pKNT25	*ori*_p15*,*_ Km^R^ *, lacp -*MCS - *cya*T25	Medium	[31]
pKT25	*ori*_p15*,*_ Km^R^ *, lacp-cya*T25*-*MCS	Medium	[31]
pKT25-zip	*ori*_p15*,*_ Km^R^ *, lacp-cya*T25*-*GCN4 leucine zipper	Medium	[31]
pLKB2	pKT25 with MCS modified	Medium	Kusiak L.^2^
pLKB4	pUT18C with MCS modified	High	Kusiak L.^2^
pMPB13.3	*ori*_RA3_, Km^R^ pABB20-*lacI* ^q^ *-tacp-nic*	Low	Przyłuski M.^2^
pMPB13.4	*ori*_RA3_, Km^R^ pABB20-*lacI* ^q^ *-tacp-mobC-nic*	Low	Przyłuski M.^2^
pPT01	*ori*_SC101_, Km^R^, promotorless *xylE*	Medium	[50]
RA3	BHR^1^, IncU, Cm^R^_,_ Sm^R^_,_ Su^R^	Low	Hayes F.^3^
pUC18	*ori*_MB1_, Ap^R^	High	[23]
pUT18	*ori*_ColE1*,*_ Ap^R^ *, lacp-*MCS*-cyaT18*	High	[31]
pUT18C	*ori*_ColE1*,*_ Ap^R^ *, lacp -cyaT18-*MCS	High	[31]
pUT18C-zip	*ori*_ColE1*,*_ Ap^R^ *, lacp -cyaT18-*GCN4 leucine zipper	High	[31]
**Plasmids constructed in this study**			
**Designation**	**Description**		
pJSB2.1	pUC18-*mobC,* EcoRI-SalI fragment amplified by PCR with the use of primers #1 and #2 (RA3 coordinates 9837-10455 nt)		
pJSB2.2	pUC18-*mobC1-129*, EcoRI-SalI fragment amplified by PCR with the use of primers #1 and #2 spontaneous stop codon mutation at position 10225 nt (RA3 coordinates 9837-10455 nt)		
pJSB2.9	pUC18-*mobCp*, 417 bp SphI-BamHI fragment amplified by PCR with the use of primers #3 and #8 (RA3 coordinates 9435-9852 nt)		
pJSB2.11	pUC18 with 116 bp SphI-BamHI PCR fragment amplified by PCR with the use of primers #6 and #8 (RA3 coordinates 9736-9852 nt)		
pJSB2.12	pUC18 with 116 bp SphI-BamHI PCR fragment amplified by PCR with the use of primers #5 and #8 (RA3 coordinates 9736-9852 nt), mutation in a putative *oriT* motif		
pJSB2.13	pUC18 with 100 bp SphI-BamHI PCR fragment amplified with the use of primers #5 and #4 (RA3 coordinates 9736-9836 nt), mutation in a putative *oriT* motif		
pJSB2.14	pUC18 with 100 bp SphI-BamHI PCR fragment amplified with the use of primers #6 and #4 (RA3 coordinates 9736-9836 nt)		
pJSB2.15	PCR mutagenesis of pJSB2.9 with the use of primers #9 and #10 (mutVI)		
pJSB2.16	PCR mutagenesis of pJSB2.9 with the use of primers #13 and #14 (mutVII)		
pJSB2.17	PCR mutagenesis of pJSB2.9 with the use of primers #11 and #12 (mutVIII)		
pJSB2.18	PCR mutagenesis of pJSB2.9 with the use of primers #15 and #16 (mutIX)		
pJSB2.30	pUC18 *mobC1-155*, EcoRI-SalI fragment amplified by PCR with the use of primers #1 and #18 carrying (RA3 coordinates 9837-10308 nt)		
pJSB2.43	pUC18 with 61 bp oligonucleotides (primer #19 and #20), oligonucleotides are inserted in SmaI site in of pUC18		
pJSB2.44	pJSB2.43 digested by NheI and EcoRI, blunt-ended with the use of Klenow fragment and self-ligated, to remove IRIV		
pJSB2.55	pJSB2.44 *oriT45*ΔO_M_ *-lacI* ^q^ *-tacp-mobC-nic,* BamHI-SalI fragment (*lacI* ^q^ *-tacp-mobC-nic*) from pMPB13.4		
pJSB2.56	pJSB2.44 *oriT45*ΔO_M_ *-lacI* ^q^ *-tacp-nic,* BamHI-SalI fragment (*lacI* ^q^ *-tacp-nic*) from pMPB13.3		
pJSB2.57	pJSB2.55 *oriT45*ΔO_M_ *-lacI* ^q^ *-tacp-mobC-nic-*Tra_RA3_-*korCp-korC,* SmaI-SalI fragment (Tra_RA3_-*korCp-korC*) from pJSB1.24		
pJSB2.58	pJSB2.56 *oriT45*ΔO_M_ *-lacI* ^q^ *-tacp-nic-*Tra_RA3_-*korCp-korC,* SmaI-SalI fragment (Tra_RA3_-*korCp-korC*) from pJSB1.24		
pJSB4.1	pBBR1MCS-1 *lacI* ^q^, *tacp-mobC*, BamHI-SalI from pJSB5.1		
pJSB4.2	pBBR1MCS-1 *lacI* ^q^, *tacp-mobC1-129*, BamHI-SalI from pJSB5.2		
pJSB5.1	pGBT30 *tacp-mobC*, EcoRI-SalI fragment from pJSB6.1		
pJSB5.2	pGBT30 *tacp-mobC1-129,* EcoRI-SalI fragment from pJSB6.2		
pJSB6.1	pET28a T7*p*-*mobC,* EcoRI-SalI fragment from pJSB2.1		
pJSB6.2	pET28a T7*p*-*mobC1-129,* EcoRI-SalI fragment from pJSB2.2,		
pJSB6.30	pET28a T7*p*-*mobC1-155,* EcoRI-SalI fragment from pJSB2.30		
pJSB7.9	pPT0I *mobCp*-*xylE*, SphI-BamHI fragment from pJSB2.9		
pJSB7.10	pPT0I *mobCp*-*xylE*, 229 bp SphI-BamHI fragment PCR amplified with the use of primers #7 and #8 (RA3 coordinates 9623- 9852 nt)		
pJSB7.11	pPT0I *mobCp*-*xylE,* SphI-BamHI fragment from pJSB2.11		
pJSB7.12	pPT0I *mobCp*-*xylE,* SphI-BamHI fragment from pJSB2.12		
pJSB7.13	pPT0I *mobCp*-*xylE,* SphI-BamHI fragment from pJSB2.13		
pJSB7.14	pPT0I *mobCp-xylE,* SphI-BamHI fragment from pJSB2.14		
pJSB7.15	pPT0I *mobCp*-*xylE,* SphI-BamHI fragment from pJSB2.15		
pJSB7.16	pPT0I *mobCp*-*xylE,* SphI-BamHI fragment from pJSB2.16		
pJSB7.17	pPT0I *mobCp*-*xylE,* SphI-BamHI fragment from pJSB2.17		
pJSB7.18	pPT0I *mobCp*-*xylE,* SphI-BamHI fragment from pJSB2.18		
pJSB8.1	pLKB4 *cyaT18-mobC*; EcoRI-HincII fragment from pJSB6.1 cloned between EcoRI-SmaI sites of pLKB4		
pJSB8.30	pJSB8.1 digested by ClaI and self-ligated to remove 3′ end of *mobC* (*mobC1-155*)		
pJSB9.1	pKGB4 *mobC-cyaT18,* EcoRI-SacI fragment amplified by PCR with primers #1 and #17		
pJSB10.1	pLKB2 *cyaT25-mobC*; EcoRI-HincII fragment from pJSB5.1 cloned between EcoRI-SmaI sites of pLKB2		
pJSB10.2	pLKB2 *cyaT25-mobC1-129*; EcoRI-HincII fragment from pJSB5.2 cloned between EcoRI-SmaI sites of pLKB2		
pJSB11.1	pKGB5 *mobC-cyaT25*; EcoRI-SacI fragment from pJSB9.1.1		

^1^BHR-broad-host-range.

^2^Institute of Biochemistry and Biophysics, Department of Microbial Biochemistry, Polish Academy of Sciences.

^3^Faculty of Life Sciences and Manchester Interdisciplinary Biocentre, The University of Manchester, Manchester, UK.

The high-copy number expression vector pGBT30 [35], based on the pMB1 replicon with *lacI*^q^ and *tacp* was used for regulated expression of *mobC* derivatives. The *tacp-mobC* transcriptional fusion with *lacI*^q^ was re-cloned also into the medium copy number broad-host range vector pBBR1MCS-1 [36].

The mutant *mobC1-129* allele resulted from a spontaneous nucleotide substitution introducing a stop codon after A129 of MobC. The deletion mutant *mobC1-155* was constructed by cleavage of pJSB8.1 by ClaI, filling-in the 5′ overhangs and re-ligation. This led to the N-terminal 155 amino acids from MobC being extended by three residues. Plasmids for over-expression and purification of the MobC derivatives were constructed by inserting the *mobC* variants as EcoRI-SalI fragments into pET28a Km^R^ (Novagen).

### Site-directed mutagenesis *in vitro*

To introduce mutations in the *oriT-mobCp* region an *in vitro* PCR- based site-directed mutagenesis method (Stratagene) was used with the high fidelity PfuTurbo DNA polymerase. Pairs of complementary primers #9/#10, #11/#12, #13/#14 and #15/#16 (Additional file 1) were designed to introduce nucleotide substitutions in a particular region of the amplified plasmid DNA accompanied by either removal of an existing restriction site or introduction of a new one to facilitate screening. Candidate mutant plasmids were tested for the presence/absence of the restriction site affected by the mutagenic primers and the correctness of mutagenesis was verified by sequencing.

### Bacterial transformation

Competent cells of *E. coli* were prepared by standard CaCl_2_ method [33].

### Determination of catechol 2,3-dioxygenase activity (XylE)

XylE activity (the product of *xylE*) was assayed in extracts from logarithmically growing cultures . The overnight cultures were used to inoculate 25 ml of L-broth (dilution 1:50) supplemented with antibiotics and 0.5 mM IPTG when needed. Cultures were grown for 1.5 h to 3 hrs at 37°C, centrifuged and pellets were re-suspended in 500 μl of 0.1 M KPi buffer (pH 7.5) and 50 μl of acetone and left on ice. After sonication the extracts were cleared by centrufugationat 16000× g for 15 min at 4°C. XylE activity was assayed spectrophotometrically according to Zukowski method [37]. The reaction was initiated by addition of 0.2 mM catechol solution. One unit of catechol 2,3- dioxygenase activity is defined as the amount of enzyme needed to convert 1 μmol of catechol to 2-hydroxymuconic semialdehyde in 1 minute per mg of protein. Protein concentration was determined using the Bradford method [38].

### Purification of His_6_-tagged MobC derivatives

For protein over-production and purification, *E. coli* BL21(DE3) was transformed with pET28 derivatives encoding N-terminally His_6_-tagged MobCs. The overnight inoculum of the transformant was diluted 1:50 to 500 ml of L-broth with kanamycin and cultured with shaking at 37°C for 1.5 hours. Then the 0.5 mM IPTG was added and culture left to grow for two hours. The cells were pelleted by centrifugation, suspended in 1 ml of sonication buffer (50 mM sodium phosphate pH 8.0, 300 mM NaCl), and disrupted by sonication. The extract was purified by affinity chromatography as described previously [39] with the use of Protino column (Macherey-Nagel). Eluted protein fractions were analyzed by SDS-PAGE using a PHAST system (Pharmacia) with 20% homogeneous gels.

### Analysis of protein-DNA interactions by electrophoretic mobility shift assay (EMSA)

PCR- amplified DNA fragments (417 bp) of modified variants of the *oriT-mobCp* region were excised from agarose gels and purified using the Gel-Out kit (A&A Biotechnology). The protein-DNA binding reactions were performed for 15 min at 37°C in binding buffer 25 mM Tris-HCl pH 8.0; 10 mM MgCl_2_; 50 mM NaCl; 0.1 mg ml^-1^ BSA) in a final volume of 20 μl with increasing amounts of His_6_-MobC added. The MobC binding was analyzed on 1.2% agarose gels run in 1xTBE buffer. The gels were stained with ethidium bromide and DNA visualized under UV light.

### Cross-linking with glutaraldehyde

His_6_-tagged MobCs purified on Ni^2+^-agarose column were cross-linked with glutaraldehyde [39] and separated on 20% (w/v) SDS-PAGE gel. The proteins were transferred onto a nitrocellulose membrane and Western blotting with anti-His tag antibodies was performed as described previously [40].

### Conjugation/ mobilization procedure

In the mobilization experiments the donor strain DH5α carried the RA3 or pJSB1.24 (pBGS18 [41] derivative with Tra_RA3_ module and *korC* gene; [42] as the helper plasmid and the mobilizable pPT01 derivatives with the variants of *oriT*-*mobCp* region inserted. In conjugation experiments strains: DH5α(pUC18), DH5α(pJSB2.57), DH5α(pJSB2.58)(pBBR1MCS-1), DH5α(pJSB2.58)(pJSB4.1), DH5α(pJSB2.58)(pJSB4.2) were used as donors.

DH5α Rif^R^ strain was used as the recipient. Aliquots of 100 μl of overnight cultures of the donor and recipient strains were mixed (1:1) and incubated on L-agar plates for 2 hours at 37°C. Cells were scrapped, re-suspended in L-broth and 10 μl aliquots of serial 10-fold dilutions were spotted onto L-agar plates with 100 μg ml^-1^ rifampicin and 50 μg ml^-1^ kanamycin to estimate the number of transconjugants. In parallel, 100 μl of the donor strain overnight culture was incubated on L-agar plate for 2 hours at 37°C, cells were scrapped, diluted and plated on L-agar or L-agar with antibiotics selective for the donor strain. The transfer frequency was calculated as the number of transconjugants per donor cell.

### Bacterial adenylate cyclase two-hybrid system (BACTH system)

The dimerization of MobC *in vivo* was analyzed using the bacterial adenylate cyclase two-hybrid (BACTH) system in *E. coli* [31]. The MobC protein was fused translationally to CyaT18 fragment and to CyaT25 fragment at the N- or C-terminus using two pairs of compatible vectors (pUT18/ pKNT25 and pUT18C/ pKT25). *E. coli* BTH101, an adenylate cyclase deficient strain (*cya*), was co-transformed with the appropriate pairs of BACTH plasmids and plated on MacConkey medium suplemented with 1% (w/v) maltose, 0.5 mM IPTG and selective antibiotics. The plates were incubated for 48 h at 27°C. The ability to ferment maltose indicated the CyaA reconstitution through interactions between the fused polypeptides.

## Results

### MobC is an autorepressor of the *mobC-nic* operon

The transfer module of RA3 (coordinates 9400-32300 nt) is located between the stability module and class I integron [21]. The first conjugative transfer operon, bi-cistronic *mobC-nic,* preceded directly by *oriT*, encodes MobC and relaxase Nic (Figure 1A).

The homologs of MobC from IncU plasmids (Figure 2B) have been found encoded in the promiscuous plasmids from PromA group [25], by loci positioned in the junction region between maintenance and conjugative transfer operons that display similar genetic organization as IncU plasmids [43]. The presence of the highly conserved structural motif LxxugxNlNQiaxxLn in the C- terminal part classifies MobC and its PromA homologs [44–46], as the putative conjugative transfer auxiliary proteins of MOB_P_ relaxases [13].

MobC_RA3_ is a putative DNA binding protein of 176 amino acids with predicted ribbon-helix-helix (RHH) motif (Figure 2A) according to the primary sequence analysis [47] and secondary structure modelling (I-TASSER, Additional file 2). A characteristic pattern of alternating hydrophilic-hydrophobic side chains is present along N-terminal β-strand with hydrophobic side chains at conserved positions 3, 5, and 7 (Figure 2A) while a positively charged residue usually occurs at position 2 or 6 (in MobC Arginine is present at position 6). A second feature of the RHH motif is a conserved G-X-S/T/N sequence in the loop between helix α1 and helix α2 (GFT in MobC). At least four hydrophobic residues are usually present in helix α1 and helix α2 that together with hydrophobic side chains at positions 3, 5, and 7 comprise the hydrophobic core of RHH motif [47]. In the MobC there are six hydrophobic residues in the predicted helices.

The RHH superfamily of prokaryotic DNA binding factors [47], encompasses several transfer auxiliary proteins like TrwA of plasmid R388 (MOB_F_), TraY of plasmid F (MOB_F_), MbeC of ColEI (MOB_HEN_) and NikA of R64 (MOB_P_) [13,28,48,49].

To analyze the role of MobC in regulation of *mobCp* expression the 417-bp DNA fragment upstream of the *mobC*_RA3_ gene (Figure 1B) was cloned into a promoter-probe vector pPT01 [50] in front of the promoterless *xylE* cassette to construct pJSB7.9. Measurements of catechol 2,3-dioxygenase activity (XylE) in the extracts of DH5α(pJSB7.9) cells from the exponential phase of growth demonstrated a moderate level of *mobCp* transcriptional activity (0.5 U of XylE).

The *mobC* orf was cloned into the high-copy number expression vector pGBT30 [35] under the control of *tacp* (synthetic IPTG- inducible promoter), to construct pJSB5.1. Introduction of pJSB5.1 into the DH5α(pJSB7.9 *mobCp-xylE*) strain led to 20-fold repression of *mobCp* even without MobC over-production indicating that MobC is a very potent repressor. Induction of MobC synthesis in DH5α(pJSB7.9)(pJSB5.1) by culturing in the presence of 0.5 mM IPTG for two hours switched off *mobCp* completely (<0.002 U). No decrease in XylE activity was observed when strain DH5α(pJSB7.9)(pGBT30) was grown in the presence of IPTG.

### Mapping of MobC operator

**Figure 3:**
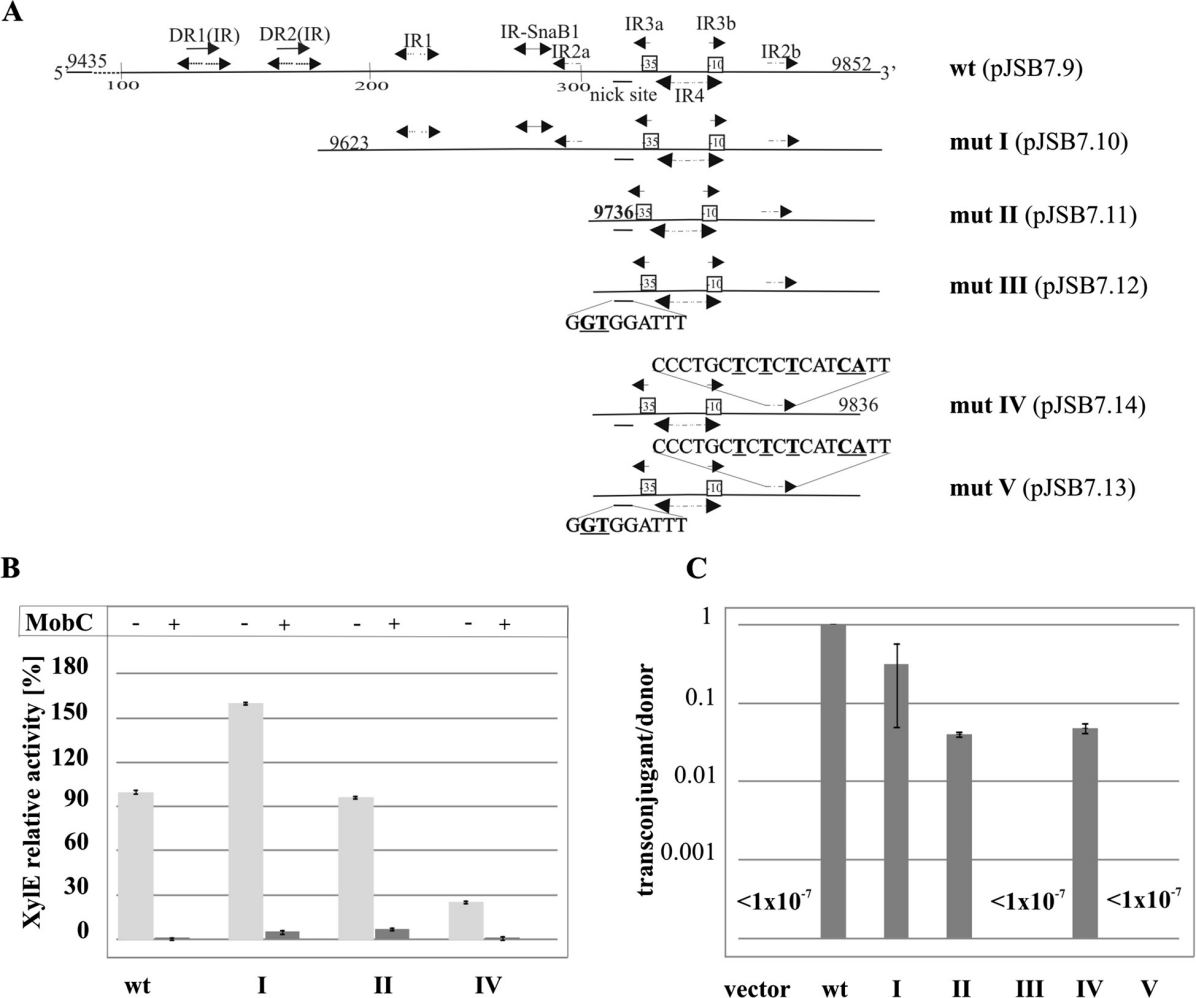
**Deletion mapping of MobC binding site in the** ***mobCp*** **region. A**. Schematic representation of deletion and point mutation derivatives. Coordinates of RA3 fragments used in the analysis and structural motifs identified in the region are shown. Sequences of modified motifs (nick site and IR2b) are shown with the modifications underlined and in bold. **B**. MobC regulation of *mobCp in vivo*. DH5α strains carrying pJSB7.9 (*mobC*_*p-*_ *xylE*) or *mobCp* mutant derivatives (labeled according to scheme **A)** were transformed with empty expression vector pGBT30 or with pJSB5.1 *tacp-mobC*. Diagram presents XylE activities assayed in extracts of the various double transformants relative to the XylE activity detected in extract of control strain DH5α(pJSB7.9)(pGBT30). Light grey bars correspond to the results obtained for the double transformants with derivatives of pPT01 and the empty vector pGBT30, dark grey bars demonstrate results obtained for thestrains with the same pPT01 derivatives but with pJSB5.1 *tacp-mobC*. Mean values with standard deviation of at least three assays are shown. **C**. DH5α(RA3) strain was transformed with pPT01 derivatives carrying deletion variants of *mobCp* region. Double transformants were used as donors in conjugation with DH5α Rif^R^ strain as the recipient. The frequency of mobilization is indicated on a semi-logarithmic scale as the number of transconjugants/donor cells, where *vector* corresponds to empty pPT01, *wt* to pJSB7.9, and *roman numerals* to the mutants presented in panel **A**. Mean values with standard deviation of at least three experiments are shown.

The *mobCp* sequences have been postulated previously as the TTGCTA and TAAAAT hexamers separated by 20 nt [43], motifs close to the consensus -35 and -10 sequences recognized by RNAPσ^70^ (Figure 1B). The sequence corresponding to the putative nick site of *oriT* [21] maps 50 nt upstream of the predicted transcription start point (tsp) for *mobC*. The analyzed region of 417 bp contains numerous structural motifs: two direct repeats DR1(IR) and DR2(IR) overlapped by inverted repeats and five additional inverted repeats designated IR-SnaBI and IR1 to IR4 (Figure 1B). The IR-SnaBI motif was identified previously as the KorB-binding site in the *parS* region [43]. Two of the palindromic sequences, IR2 and IR3, have their arms separated by 75 nt and 26 nt, respectively. To map O_M_ in the *mobCp* region a series of deletion derivatives was constructed by PCR amplification and the truncated fragments with intact putative promoter sequences were cloned into the promoter-probe vector pPT01 (Figure 3A). All these derivatives were analyzed for promoter activity in the presence of empty expression vector (pGBT30) and MobC produced *in trans* (pJSB5.1 *tacp-mobC*).

Some variability in the level of *mobCp* expression was observed among the mutants analyzed (Figure 3B). Significantly, the deletion derivatives showed the same susceptibility to MobC repression as the original 417-bp fragment present in pJSB7.9 (Figure 3B). This indicated that DR1(IR), DR2(IR), IR1 and the left arm of IR2 (IR2a) were not required for MobC to exert its regulatory effect. To exclude the possibility that one arm of IR2 could suffice for MobC binding, IR2b was mutated in a truncated promoter fragment of 100 bp (pJSB7.14) by site-directed PCR mutagenesis. The deletion of the IR2a arm and multiple substitutions in the remaining IR2b arm had no significant influence on MobC repression.

**Figure 4:**
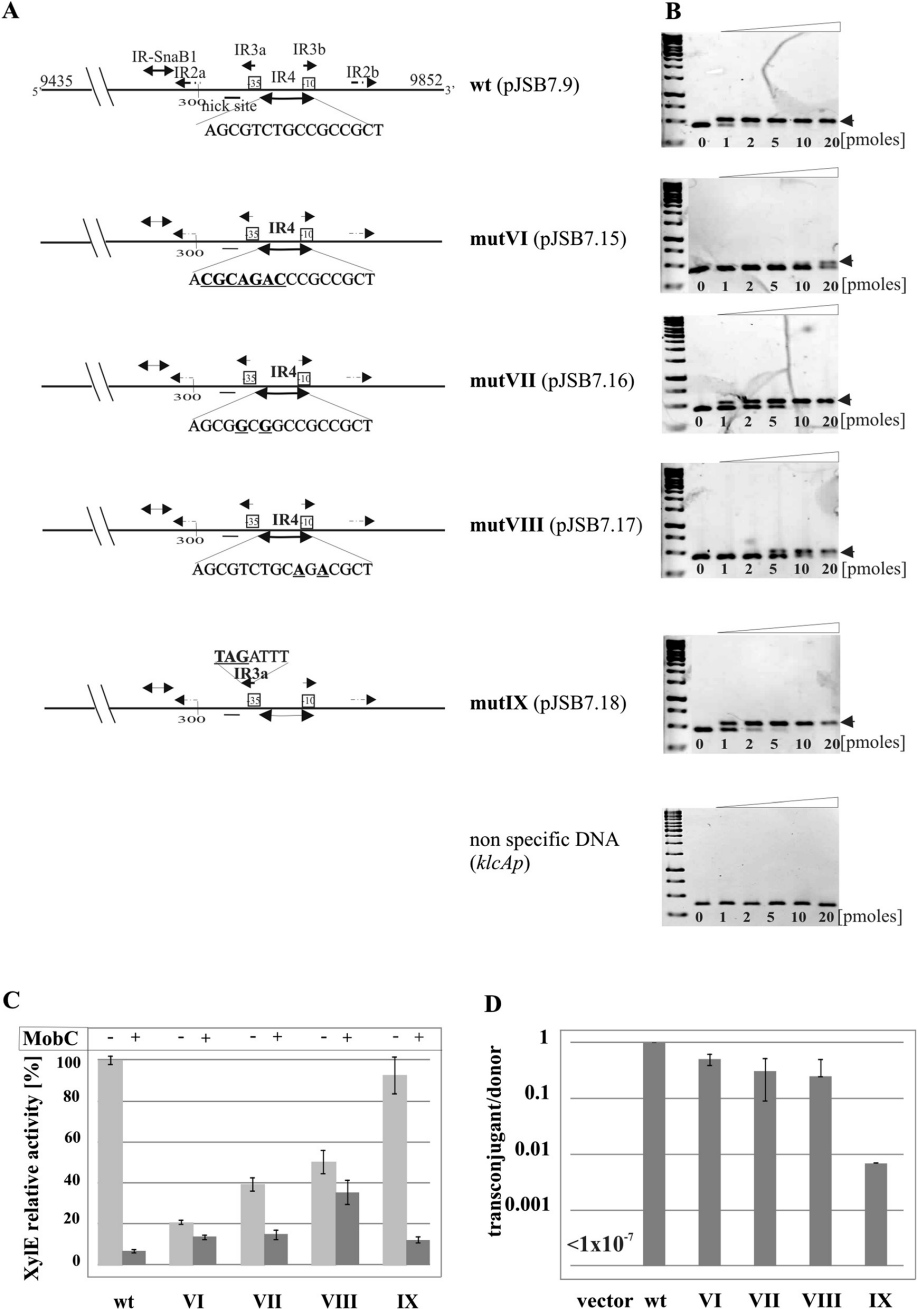
**Modifications of MobC operator. A**. Scheme of PCR site-directed modifications in *mobCp* region. Sequences of modified motifs (IR3 and IR4) are shown with the modifications underlined and in bold. Mutant alleles (mutVI-IX) were PCR-amplified and analyzed in EMSA (panel **B**), cloned into promoter-probe vector pPT01 upstream of *xylE* cassette (panel **C** and **D**). **B**. DNA binding activity of MobC *in vitro*. Two picomoles of 417-bp DNA fragments amplified by PCR on templates shown in panel **A** were incubated with 0 to 20 picomoles of His-tagged MobC in 20 μl of binding buffer at 37°C for 15 minutes. The complexes were separated on 1.2% agarose gels run in 1xTBE and visualized by ethidium bromide staining. The bottom panel refers to EMSA of MobC with unspecific DNA fragment, PCR-amplified *klcAp* of RA3 (coordinates 2336-2704 nt). **C**. MobC regulation of *mobCp* derivatives *in vivo*. DH5α strains carrying pJSB7.9 (wt 417 bp fragment) or its mutant derivatives (labeled according to panel **A**) were transformed with empty vector pGBT30 (light grey bars) or pJSB5.1 *tacp-mobC* (dark grey bars). Diagram presents XylE activities in extracts of the double transformants relative to the XylE activity detected in extract of DH5α(pJSB7.9)(pGBT30) strain. Mean values with standard deviation of at least three assays are shown. **D**. DH5α (RA3) strain was transformed with pPT01 derivatives carrying substitution mutant variants of *mobCp* region. Double transformants were used as donors in conjugation with DH5α Rif^R^ strain as the recipient. The frequency of mobilization is indicated on a semi-logarithmic scale as the number of transconjugants/donor cell, where *vector* corresponds to empty pPT01, *wt* to pJSB7.9 (*mobCp-xylE*), and roman numerals to the mutants presented in panel **A**. Mean values with standard deviation of at least three experiments are shown.

The above analysis of truncated *mobCp* fragments localized the MobC-binding site to a 100-bp region encompassing the promoter sequences and two palindromic motifs IR3 and IR4. Both IR3 and IR4 partly overlap the predicted promoter sequence (Figure 1B) hence only limited modifications could be introduced into this region without affecting the promoter integrity (Figure 4A). The PCR-based mutagenesis was performed on the pUC18 [43] derivative with the whole 417-bp insert (pJSB2.9), since the truncated *mobCp* regions exhibited variations in the transcriptional activity. The mutated inserts were re-cloned into the promoter-probe vector pPT01 and tested for *mobCp* activity and sensitivity to the MobC, produced from pJSB5.1 *in trans*. Nevertheless, even those few nucleotide substitutions led to a decrease of the *mobCp* activity (up to 5-fold) in some of the constructs. MobC repression was unaffected when IR3a was modified as in pJSB7.18 (GCAATT → ***TAG***ATT) whereas substitutions in IR4 (pJSB7.15, pJSB7.16 and pJSB7.17) led to a decrease in repression by MobC (Figure 4C).

When one arm of the IR4 palindrome was modified (AGCGTCTG↑CCGCCGCT → A***CGCAGAC***↑CCGCCGCT) to give variant mutVI (pJSB7.15), the promoter became hardly sensitive to MobC repression (repression index 1.5), strongly suggesting that IR4 is the operator for MobC and that efficient MobC binding requires the intact palindrome (Figure 4A and C).

IR4 is not a perfect palindrome, having two pairs of non-complementary nucleotides (AGCG*T*C*T*G↑C*C*G*C*CGCT). To create a perfect palindromic sequence either left or right arm of the IR4 was modified by site-directed mutagenesis. Version of IR4 (AGCG***G***C***G***G↑CCGCCGCT) in mutVII (pJSB7.16) had a higher content of GC pairs whereas version of IR4 (AGCGTCTG↑C***A***G***A***CGCT) in mutVIII (pJSB7.17) had a higher content of AT pairs than the wt sequence. None of these perfect palindromes was fully effective in MobC binding. In comparison to the 20-fold repression observed for the native non-perfect IR4, the repression index was 1.4 and 2.6, when the mutated versions of IR4 mutVIII or mutVII, respectively, were introduced into *mobCp* (Figure 4C).

### DNA binding by MobC

The *mobC* orf was cloned under *T7p* into pET28a to give pJSB6.1. His_6_-MobC over-produced in BL21(DE3)(pJSB6.1) strain was purified by affinity chromatography and used in the Electrophoretic Mobility Shift Assays (EMSA) with wt *mobCp* fragment and its mutated versions.

MobC was able to bind and retard efficiently the 417-bp fragment comprising wt *mobCp* whereas no MobC binding was observed when an unspecific DNA fragment was used (Figure 4B). The affinity of MobC towards its operator O_M_ in the *mobCp* region estimated by K_app_ (K_apparent_ - the protein concentration at which 50% of the DNA fragments were shifted) varied in the range of 30-50 nM for different protein preparations.

DNA binding studies performed with four mutated *mobCp* fragments showed that modification of IR3 (mutIX) had no effect on the MobC binding (K_app_ ~40 nM). The binding affinity of MobC to fragments with the three versions of IR4 depended on the type of nucleotide substitution (Figure 4B and C). The multiple nucleotide changes in one arm of IR4 (mutVI present in pJSB7.15) drastically decreased the MobC affinity (K_app_ > 0.8 μM). Creating a perfect palindromic sequence by replacing two Ts with two Gs in the left arm of O_M_ (mutVII) increased the K_app_ to 100 nM. The fragment with the perfect palindromic sequence mutVIII with two Cs substituted by As in the right arm (pJSB7.17) was bound by MobC with an approximately 10-fold lower affinity (K_app_ ~ 400 nM) than the wt sequence. The data of *in vitro* experiments correlated with the decreased index of repression *in vivo* and confirmed that IR4 plays a vital role in MobC binding and as such it was designated O_M_ - operator for MobC.

### Dimerization ability of MobC

**Figure 5:**
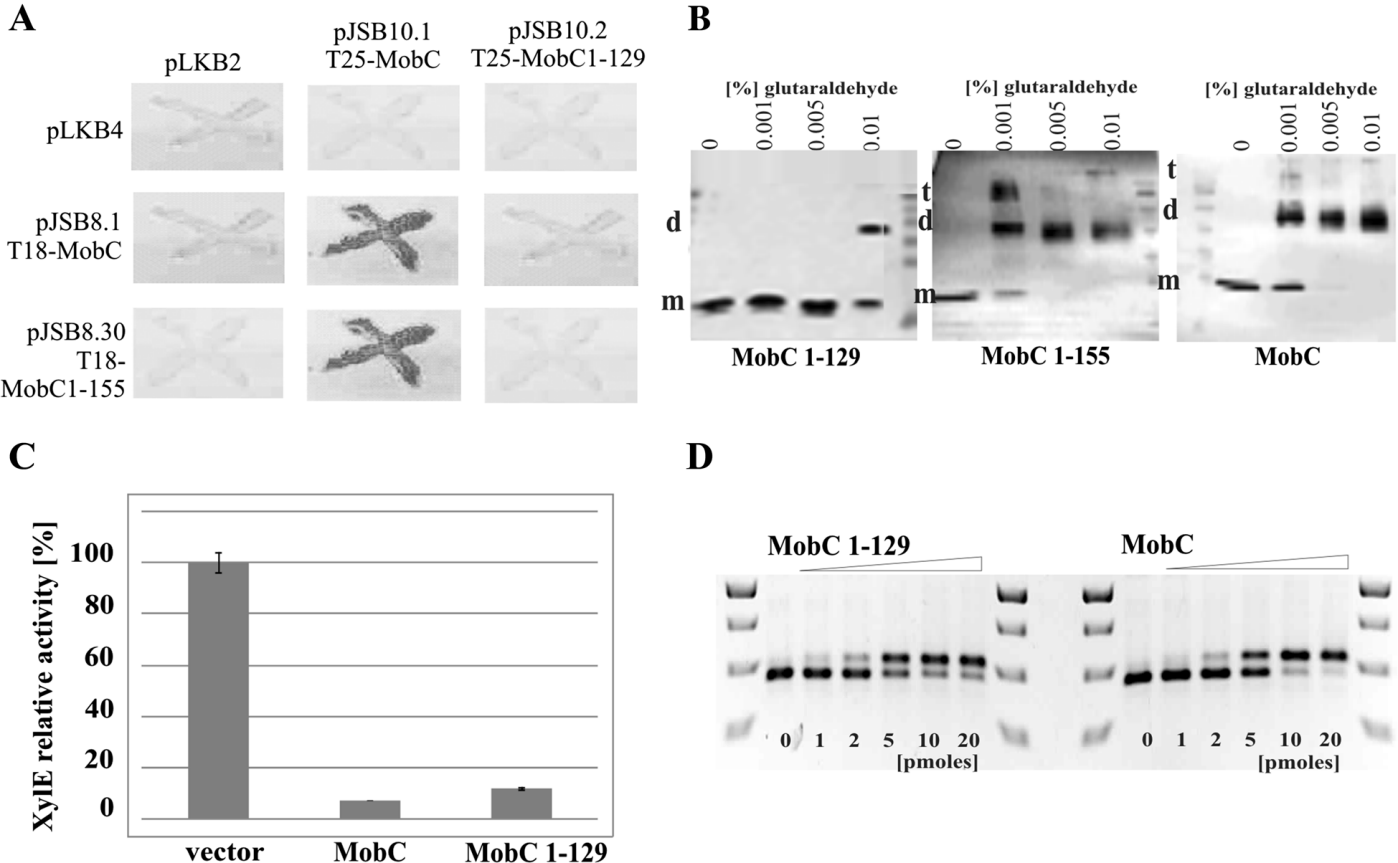
**MobC C-terminus is required for oligomerization but not DNA binding. A**. Bacterial two-hybrid system *in vivo.* The *mobC* orf was cloned into two sets of BACTH vectors to be linked with CyaA fragments by N-or C-terminus. Photographs document the ability to ferment maltose by double transformants of BTH101*cyaA* strain with plasmid encoding CyaA fragments linked to N-termini of MobC derivatives. The pLKB2 and pLKB4 represent modified empty BACTH vectors pKT25 and pUT18C, respectively. Similar results were obtained for plasmids encoding CyaA fragments linked to C- termini of MobC derivatives (data not shown). **B**. Glutaraldehyde cross-linking *in vitro*. His-tagged MobC derivatives (0.1 mg ml^-1^) were incubated with increasing concentrations of glutaraldehyde (0.001%, 0.005% and 0.01%). The complexes were separated on 20% polyacrylamide gels by SDS-PAGE, transferred onto a nitrocellulose membrane and visualized byWestern blotting with anti-His tag antibodies. Bands corresponding to monomeric (**m**), dimeric (**d**) and terameric (**t**) form of the MobC variants analyzed are marked. The protein migrating faster than MobC1-129 is probably a degradation product. **C**. Regulation of *mobCp* by truncated MobC1-129. Allele *mobC1-129* was cloned into pGBT30 under *tacp* (pJSB5.2) and introduced into DH5α(pJSB7.9) strain. The double transformant strains were grown without IPTG. The XylE activity is expressed relative to the activity detected in DH5α(pJSB7.9)(pGBT30) strain. DH5α(pJSB7.9)(pJSB5.1 *tacp-mobC*) strain was used as the control. Mean values with standard deviation of at least three assays are shown. **D**. DNA binding by MobC1-129 *in vitro*. Two picomoles of 417-bp DNA fragment containing *mobCp* were incubated with increasing quantities (0 to 20 picomoles) of His-tagged MobC derivatives in 20 μl of binding buffer at 37°C for 15 minutes. The complexes were separated on 1.2% agarose gels run in 1xTBE and visualized by ethidium bromide staining.

To analyze the ability of MobC to self-interact *in vivo* and to define the role of the C-terminus in this process, two 3′-end deletion mutant derivatives *mobC1-129* and *mobC1-155* were constructed, encoding N-terminal 129 and 155 amino acids, respectively (Figure 2B). The *mobC* orf and its mutant derivatives were cloned into the vectors of bacterial two-hybrid system BACTH [31]. The wt *mobC* was inserted into two pairs of BACTH vectors to fuse MobC translationally with Cya fragments at the N- or the C-terminus. In two-hybrid tests MobC demonstrated a dimerization ability independently of which end of the protein was linked to the Cya fragments. The two truncated forms of MobC differed in their ability to self-interact in the BACTH system (Figure 5A). MobC1-155 was not impaired in the dimerization *in vivo* whereas MobC1-129 lacked the self-interaction ability, suggesting that the C-terminal residues between 130 and 155 are important for this interaction (Figure 2B).

The *mobC1-129* and *mobC1-155* orfs were cloned under *T7p* into pET28a to give pJSB6.2 and pJSB6.30, respectively. The His_6_-MobC1-129 and His_6_-MobC1-155 were over-produced in BL21(DE3) transformants and purified by affinity chromatography according to the protocol used for WT MobC.

Glutaraldehyde cross-linking confirmed the ability of WT MobC and MobC1-155 to form dimers, trimers and tetramers in solution (Figure 5B) at low concentrations of the protein and the cross-linking agent. MobC1-129 only formed dimers under the highest glutaraldehyde concentration used. This suggests that the C-terminus of MobC RA3 is not absolutely required for dimerization, but its presence probably stabilizes the dimers (interactions between MobC1-129 and WT MobC were too weak to be detected in the BACTH system) and probably facilitates the formation of higher oligomers. To verify these results the gel filtration chromatography was used to analyze the oligomeric state of purified proteins WT MobC and MobC1-129. The molecular weights of both proteins in their monomeric state were estimated by mass spectrometry as 24 kDa and 18 kDa, respectively. The gel filtration profile of WT MobC indicated the major form of 49 kDa (Additional file 3) suggesting that MobC exists as a dimer in solution. No higher oligomers of MobC were detected under used conditions. The gel filtration profile of MobC1-129 confirmed its ability to form dimers since the major peak corresponded to protein of 42 kDa.

Purified His-tagged MobC1-129 was used in EMSA with wt *mobCp* fragment and found to bind DNA with a similar affinity as WT MobC (Figure 5D).

To confirm the ability of MobC1-129 to bind DNA *in vivo* the deletion variant *mobC1-129* was cloned under *tacp* into the pGBT30 expression vector (pJSB5.2). Using the *mobCp-xylE* transcriptional fusion in the two-plasmid regulatory system we found that MobC1-129 retained activity of a potent repressor. Strong repression of *mobCp*-*xylE* was observed even at a low concentration of the truncated MobC1-129 *in trans* (when no IPTG inducer was added), similarly to the effect of WT MobC. This suggests that the observed defect in dimerization (BACTH) does not affect the repressor function of MobC1-129 *in vivo* (Figure 5C).

### Frequency of mobilization of modified *mobCp* fragments by RA3 conjugative system

In other studied conjugative systems auxiliary proteins are essential as being involved in relaxosome formation, specific recognition of nick site by relaxase and *oriT* processing [5]. To check if binding of MobC to *oriT*_RA3_ affects basic relaxase function, conjugative transfer mobilization experiments with plasmids carrying *mobCp* derivatives were performed.

The pPT01 plasmid with a wt *mobCp* fragment inserted (pJSB7.9) was introduced into the DH5α(RA3) strain to create a donor strain that could transfer RA3 by conjugation and/or mobilize pJSB7.9 into the recipient strain DH5α Rif^R^. The frequency of RA3 self-transfer was estimated to be close to 100% (one transconjugant per donor cell), the same as the mobilization frequency of pJSB7.9 by RA3. Similar experiments performed with the truncated derivatives of *mobCp* demonstrated that deletions of the upstream sequences decreased the mobilization frequency 5 to 20-fold (Figure 3C).

In the course of this analysis we also constructed mutant derivatives with two nucleotide substitutions at the putative nick site (mutIII and mutV as shown on Figure 3A). pPT01 derivatives carrying these alleles (pJSB7.12 and pJSB7.13) were not transferable by RA3 to the recipient strain under the conditions used (estimated frequency of transfer < 1 per 10^7^ donor cells). These results confirm the vital role of these two nucleotides in the recognition/nicking of *oriT* (Figure 3C).

A 100-fold decrease in the mobilization frequency was observed for a derivative of the 417-bp *mobCp-oriT* fragment with several substitutions in the left arm of IR3 (pJSB7.18, mutIX) (Figure 4D). Further studies will be necessary to define the role of these nucleotides in the binding of relaxase to *oriT*.

Most significantly, modifications of O_M_ in a full-length *mobCp-oriT* fragment (417 bp) did not drastically affect the mobilization frequency of tested pPT01 derivatives (Figure 4D). This strongly implicates that the MobC binding to DNA at O_M_, although important for the transcriptional control of *mobCp*, is not strictly required for the conjugation process. It was further confirmed in the studies described in the next paragraph (for pJSB2.57).

Although the data clearly demonstrated that MobC-DNA interactions are not important in the mobilization experiments (when autoregulatory function of MobC is irrelevant) it did not exclude the possibility of MobC acting as the auxiliary protein through direct interactions with the relaxase or other components of the conjugative machinery.

### Influence of MobC on conjugation frequency of vectors with Tra module of RA3

The conjugative transfer module of RA3 (coordinates 9437-22925 nt) was previously cloned into the high-copy-number plasmid pBGS18 [41] together with *korC* gene (coordinates 3391-3705 nt) encoding RA3 global transcriptional regulator [42,51]. Such construct pJSB1.24 was capable of self-transmission [42] at the frequency comparable with parental RA3 (approximately one transconjugant per donor cell).

**Figure 6:**
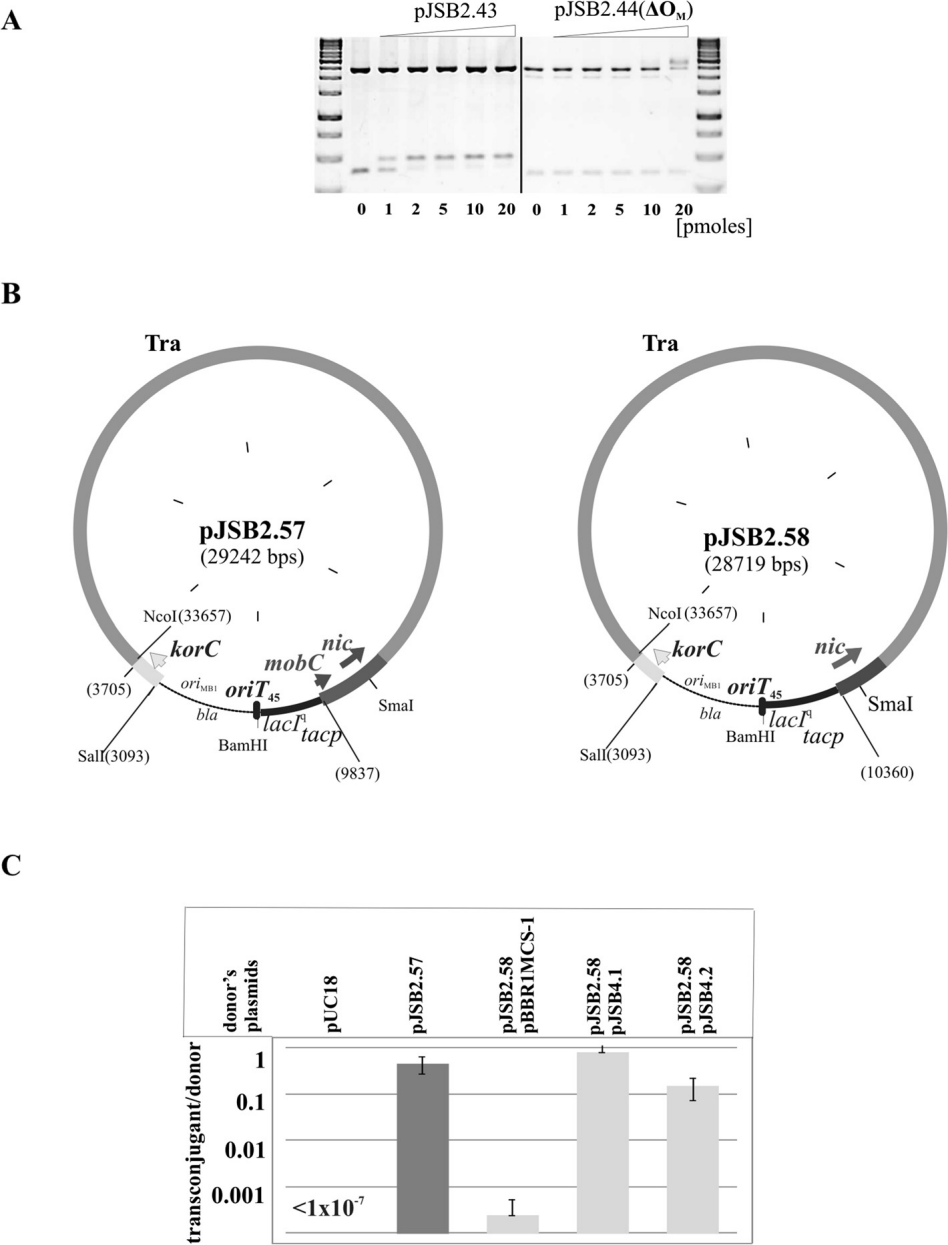
**MobC influence on efficiency of self-transmission of plasmids with RA3 conjugative module. A**. DNA binding of MobC *in vitro.* Plasmid DNAs of pJSB2.43 (61 nt insert with O_M_) and pJSB2.44 (45 nt insert without O_M_) were digested by PvuII and incubated with different amounts of His_6_-MobC in 20 μl of binding buffer at 37°C for 15 minutes (0-20 pmoles). The complexes were separated on 1% agarose gels run in 1xTBE and visualized by ethidium bromide staining. Fragment 2364 nt corresponds to “unspecific” vector DNA, small fragments in both plasmid DNAs contain *oriT*_RA3_ with O_M_ (383 nt) or without MobC binding site (367 nt). **B**. Schematic presentation of tested vectors carrying the RA3 conjugative module with *mobCp* substituted by *lacI* ^q^ *tacp* (no O_M_). Dark grey regions correspond to the contiguous Tra_RA3_ region and light grey parts indicate *korCp-korC* fragment from RA3 maintenance module. Black regions show *mobC-nic* or *nic* genes under *tac* promoter control. The 45 nt o*riT*_RA3_ is indicated by black rectangular. Restriction sites used during construction of pJSB2.57 and pJSB2.58 are listed. Numbers in brackets correspond to RA3 coordinates. **C**. The effect of MobC presence on the conjugation frequency. The strains: DH5α(pUC18), DH5α(pJSB2.57), DH5α(pJSB2.58)(pBBR1MCS-1), DH5α(pJSB2.58)(pJSB4.1) and DH5α(pJSB2.58)(pJSB4.2) served as donors in conjugation with the recipient strain DH5α Rif^R^. Conjugation frequency is indicated on the semilogarithmic scale as the number of transconjugants per total number of donor cells. Mean values with standard deviation of at least three experiments are shown.

To analyze the significance of MobC for the efficiency of the conjugation process it was necessary to separate its autorepressor function from the potential conjugative transfer accessory role. The conjugative transfer region (and *korC*) was re-cloned into pUC18 and the promoter region of *mobC-nic* operon was replaced by *lacI*^q^*-tacp* regulatory region from pGBT30 [35]. The 45 nt fragment encompassing *oriT*_RA3_ but with O_M_ and part of the *mobCp* sequences deleted (coordinates 9722-9766 nt, Figure 1B), designated *oriT*_45_, was inserted into pUC18 derivative with the conjugative transfer module and *mobC-nic* under control of *tacp* to get the pJSB2.57 (Figure 6B). The frequency of self-transmission of pJSB2.57 was very high, comparable to RA3 (Figure 6C) confirming that **a/** level of expression of *mobC-nic* operon from the uninduced *tacp* is adequate for efficient conjugation process, **b/** the lack of MobC binding site O_M_ has no influence on the transfer efficiency.

To verify inability of MobC to bind to *oriT*_45_ two oligonucleotides 61 nt and 45 nt long were cloned into pUC18 vector to obtain pJSB2.43 and pJSB2.44, respectively. The 61 nt fragment encompassed *oriT*, IR3a and IR4 (O_M_) sequences (Figure 1B) whereas 45 nt fragment (as described above) was deprived of O_M_. Plasmid DNAs were cut by PvuII in two fragments. The smaller fragments of both plasmid DNA: 383 nt and 367 nt contained inserts of 61 nt and 45 nt, respectively. In the EMSA experiment the 45 nt sequence, deprived of O_M_, has not been recognized by MobC, the protein has bound specifically only to the fragment of 383 nt with 61 nt of *oriT* inserted (Figure 6A).

To check if MobC itself plays a role in the conjugative transfer the pJSB2.58 was constructed, the variant of pJSB2.57 in which the *mobC* gene was absent and only *nic* was expressed from the *tacp* (Figure 6B). The 1000-fold decrease in the frequency of self-transmission of pJSB2.58 indicated that MobC indeed acts as the conjugative transfer auxiliary protein. The strain DH5α(pJSB2.58) was transformed with medium-copy number expression plasmid pJSB4.1 (*tacp-mobC*) and the double transformant was used as the donor strain in the conjugation. The high frequency of self-transmission of pJSB2.58 was restored (Figure 6C). Similar effect of restoration was achieved when lack of *mobC* in pJSB2.58 was complemented by *tacp-mobC1-129* expressed *in trans* in the DH5α(pJSB2.58)(pJSB4.2) strain. It indicated that despite dimerization/ oligomerization deficiency MobC1-129 is not only a potent auto-repressor but also its function in enhancing the frequency of transfer is unaltered.

## Discussion

The RA3 plasmid, the archetype of IncU group, has a mosaic-modular structure with the conjugative system similar to that found in many promiscuous environmental plasmids, some of them recently classified into the new group designated PromA [25].

We have initiated an experimental dissection of the IncU conjugative transfer system to understand the reasons of the wide spreading of such modules among conjugative plasmids of different incompatibility groups and their high efficiency of transfer between a broad range of hosts. In this study we concentrated our efforts on a functional analysis of the MobC protein (encoded by the first gene of the conjugative module) and its interactions with DNA.

The data demonstrates that MobC is a potent repressor of *mobCp* and as such controls the level of relaxase production*.* Using different experimental approaches we identified the MobC-binding site O_M_ at a region overlapping the *mobC* promoter*.* This non-perfect palindromic sequence AGCGTCTG↑CCGCCGCT is recognized by MobC *in vitro* with a high affinity. Attempts to improve the O_M_ by creating a perfect palindrome failed, showing clearly that the existing slightly imperfect configuration is optimal for MobC binding.

Purified His_6_-MobC forms mainly dimers as it was shown by gel filtration chromatography in these solution conditions. Some auxiliary proteins e.g. TrwA of R388 exist as tetramers in solution [28] others like MbeC of ColE1 plasmid are mainly dimeric [13]. To localize dimerization domain we analyzed C-terminally truncated MobC derivatives. Deletion of C-terminal 47 amino acids (MobC1-129) but not 21 amino acids (MobC1-155) impaired self-interactions *in vivo* when tested in BACTH system. The purified MobC1-129 formed dimers in solution as shown by use of the molecular sieve. The intact MobC had an ability to form trimers and tetramers in the presence of the cross-linking agent whereas MobC1-129 formed only dimers under these conditions. So far the function of the higher order forms of MobC is not clear.

MobC1-129 binds efficiently to the operator sequence and represses *mobCp in vivo* to the same extent as does the WT MobC. It means that the N-terminal part is sufficient for specific DNA binding and a weak dimerization whereas the C-terminus is required to stabilize the dimers.

Deletion of C-terminal 47 amino acids removes part of the conserved “bacterial mobilization motif” (Figure 2A; Additional file 2). The role of this motif in interactions of the auxiliary proteins with the conjugation machinery has been implicated [14,30]. Truncated MobC1-129 fully complemented the role of WT MobC in enhancing the conjugative transfer. Hence whether MobC assists the relaxase Nic in its functions or stimulates the coupling protein VirD4 (or any other protein) it does not require the intact motif. Direct interactions have been detected using the BACTH system neither between MobC and Nic nor MobC and VirD4 so far (data not shown).

MobC differs from known conjugative transfer auxilliary proteins since its binding to DNA close to *oriT* seems not to be strictly required for the relaxase action at *oriT.* All mobilizable plasmids with the O_M_ modified in such a way as to abolish or severely impair MobC binding (confirmed by a decreased repression *in vivo* and lower DNA binding affinity *in vitro*) demonstrated highly similar mobilization frequency when compared to plasmid pJSB7.9 with a wt fragment inserted. Moreover, the frequency of self-transmission of plasmid pJSB2.57, carrying RA3 conjugative module but with *oriT* deprived of O_M_ (Figure 6) has not been even slightly affected.

The absence of MobC did not stop the self-transmission of pJSB2.58, although it led to 1000-fold decrease in the transfer efficiency in comparison to pJSB2.57. Whereas significant, it is a much less pronounced effect than observed in other studied plasmids when deprived of the auxiliary proteins [13,19,29].

The functionality of *oriT* in the centromere-like region of the partition operon has been shown experimentally for RA3 of IncU [21]. There, the nick site precedes the transcription start point for *mobC* by 50 nt, and substitutions of two nucleotides in this sequence (pJSB7.12 and pJSB7.13) abolish the mobilization capacity of a test plasmid (with the *oriT-mobCp* fragment inserted) by the RA3 conjugation system. Our analysis of mutants with nucleotide substitutions adjacent to the nick site has revealed the importance of one arm of a short palindromic sequence designated IR3 for the processing of *oriT* (mobilization frequency). Further studies are required to understand the role of this motif in the transfer efficiency.

**Figure 7:**
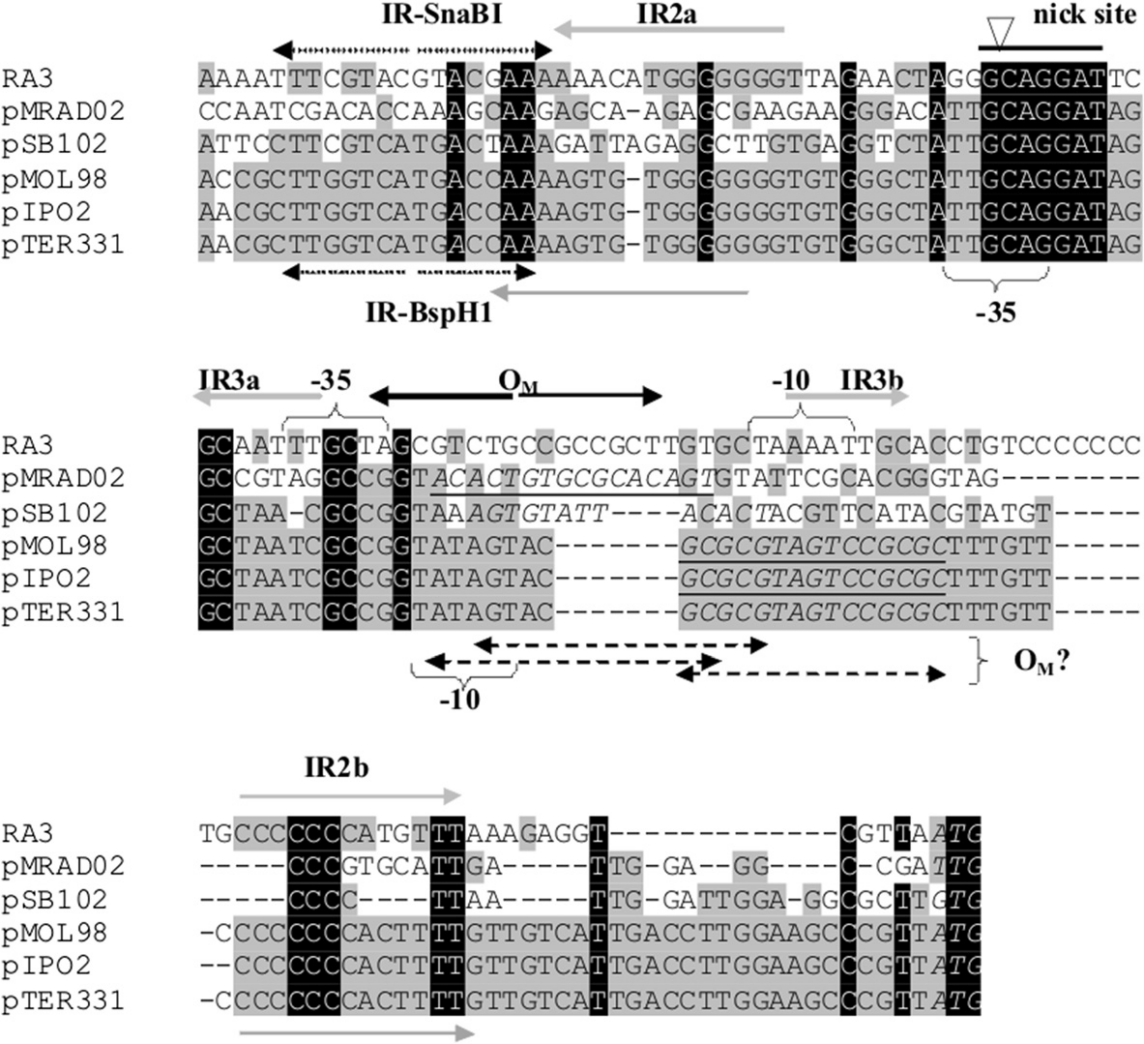
**The** ***oriT*** **regions from RA3 of the IncU group and PromA plasmids.** Alignment of DNA sequences of the *mobCp/oriT* region of RA3 ( [GenBank: DQ401103] coordinates 9703 – 9837 nt), pMRAD02 ([GenBank: CP001003.1]; coordinates 40344–40226) pSB102 ([GenBank: NC_003122]; coordinates 11373 – 11494 nt), pMOL98 ([GenBank; FJ 666348]; coordinates 45941 – 45808 nt), pIPO2 ([GenBank: NC_003213]; coordinates 11035 – 11167 nt) and pTer331 ([GenBank: EU 315244]; coordinates 10744 – 10877 nt). Identical nucleotides in all six plasmids are in white on black, those identical in three to five representatives shadowed grey. Putative promoter sequences and regulatory motifs are marked above the RA3 and below the PromA plasmid sequences. Black arrows indicate MobC binding site in RA3 identified in this work, grey arrows correspond to the arms of palindromes IR2 (GC-rich, conserved in PromA group) and IR3. Putative operators for auxiliary proteins in PromA plasmids are underlined and in italics. Start codons for homologs of MobC are in bold and italics.

Plasmids of the IncU and PromA incompatibility groups share the transcriptional organization of at least two putative conjugative transfer operons. The location of the *oriT* sequence in the promoter regions of *mobC-nic* and their counterparts in other plasmids is at the border of conjugative transfer modules and the partition operons [21,25,43–46], however the nick sites identified *in silico* in PromA representatives do not precede but rather overlap the predicted -35 sequences of promoters for the *mobC* homologs: *orf15* of pSB102, *orf21* of pIPO2, *orf57* of pMOL58, *mobC* of pTer331 and *mobC* of pMRAD02 (Figure 7).

Interestingly, the conservation of the promoter regions of the *mobC* counterparts in the IncU and PromA plasmids has been lost around the sequence of the identified O_M_ in RA3 (Figure 7). In the PromA plasmids there are palindromic sequences overlapping or located downstream of a predicted -10 motif, presumably forming the binding site for the MobC homologs.

The role of the other structural motifs in the *mobCp-oriT* region of RA3 (Figure 1B) remains unknown but a possibility of tertiary cruciform structure formation (predicted by program Geneious 6.1.3) seems very attractive especially for the two inverted repeats IR2 and IR3 with arms separated by 75 nt and 26 nt, respectively (Additional file 4).

One of these palindromes, the GC-rich IR2, is highly conserved between RA3 and three out of five PromA representatives not only in the primary sequence but also in the position of the arms encompassing promoter sequences, putative O_M_ and nick sites (Figure 7). Our future studies will be aimed at understanding the functions of these motifs and the interplay between Nic and MobC (relaxosome), IncC and KorB (segrosome) and transcriptional machinery at a potentially highly-structured *parS-oriT-mobCp* region that must accommodate all these complexes.

## Conclusions

In this work we have demonstrated that MobC of RA3 plasmid acts as an auxiliary transfer protein of dual function. It autoregulates the expression of *mobCp* controlling the level of relaxase production. It binds to DNA recognizing an imperfect palindromic sequence (O_M_) overlapping the promoter motifs. DNA binding domain is localized in the N-terminal part of the protein. The C-terminus participates in the stabilization of oligomeric forms. Besides its role as the transcriptional repressor MobC stimulates the frequency of conjugation process. For this activity MobC binding in the *oriT* region is not required. Future studies should establish whether MobC stimulates relaxosome formation, activity of relaxase or affects other stages of the conjugation process (e.g. interactions with coupling protein). The role of the structural motifs identified in the *oriT* of IncU, and conserved in putative *oriT* regions of PromA plasmids, awaits elucidation.

## Additional files

Additional file 1:
**Oligonucleotides used in this study** ^**1**^ **.**
Additional file 2:
**3D structure of MobC predicted by I-TASSER online server.** The putative RHH motif (52 aa- 99 aa) is marked red, a bacterial mobilization protein motif (117 aa-133 aa) is located mainly in helix 5 and indicated by four thin lines. The region of 19 aa deleted in MobC1-155 is colored yellow, the deletion in MobC1-129 encompasses amino acids marked yellow and green. Additional file 3:
**The gel filtration chromatography of MobC and MobC1-129.** The 0.5 ml of purified His_6_-MobC (0.24 mg ml^-1^) and His_6_-MobC1-129 (0.04 mg ml^-1^) were loaded independently on Superdex 200 10/300 GL column (GE Healthcare) equilibrated with size-exclusion chromatography buffer (10 mM Tris pH 8.0, 150 mM NaCl) with three in-line detectors: UV (ÄKTA Purifier, GE Healthcare), MALS (DAWN HELEOS-II, Wyatt Technology) and differential refractometer (Optilab T-rEX, Wyatt Technology). Data processing and molecular weight calculations were performed using ASTRA software (Wyatt Technology). Diagram presents relative units (mAU) calculated from the absorbance values of UV 280 nm. The black line corresponds to the gel filtration profile of MobC whereas the grey dashed line indicates gel filtration profile of MobC1-129. Molecular weight of complexes His_6_-MobC and His_6_-MobC1-129 are 49 kDa and 42 kDa respectively. Data from mass spectrometry show that molecular weight of His_6_-MobC monomer is 24 kDa and His_6_-MobC1-129 monomer is 18 kDa (Technical specification: Spectrometer Synapt G2 MS (Waters), LC system nanoACQUITY (Waters), Trap Column ACQUITY UPLC PrST C4 VanGuard, Pre-column 300A, 1.7 μm, 2.1 mm, 5 mm). Additional file 4:
**Single stranded DNA folding of** ***oriT***_**RA3**_ **region and its mutant derivatives.** The MobC binding site (IR4) is shadowed in grey, the -35 and -10 sequences of *mobCp* are circled in pink, the nick site is circled in red with arrows pointing the site of cleavage. The IR3 and IR-SnaBI are marked as green or blue rectangles, respectively. The mutants are designated as on Figure 4A. The DNA sequence analyzed by Geneious6.1.3 encompasses region of RA3 between 9560-9840 nt.
