# Supplementary material for: MobC of conjugative RA3 plasmid from IncU group autoregulates the expression of bicistronic mobC-nic operon and stimulates conjugative transfer
Source: BMC Microbiol. 2014 Sep 4;14:235. doi: 10.1186/s12866-014-0235-1 (PMC4175270; doi:10.1186/s12866-014-0235-1)
Supplement: Additional file 1: — Oligonucleotides used in this study 1 . [file 12866_2014_235_MOESM1_ESM.docx]

**Additional file 1: Oligonucleotides used in this study^1^.**

| **Designation** | **Sequence** |
| --- | --- |
| 1 - *mobC1* | CGGAATTC**ATG**GCAAAGAGCTATCGGATCG |
| 2 - *mobC2* | CGGTCGACTCGCTTAACTCGGCCTTTCA |
| 3 - *sphmob* | GCGCATGCTTTTCTCGTTGGAGGGTGAT |
| 4 - *inc230DM* | GCGGATCCTAACGACCTCTTTAATGATGAGAGAGCAGGG |
| 5 - *oriTM* | GCGCATGCTTAGAACTAGGGGTGGATTC |
| 6 - *oriT* | GCGCATGCTTAGAACTAGGGCAGGATTC |
| 7 - *inc230L* | TCGCATGCAAGTTAGGGGAAGCCGACGA |
| 8 - *inc230P* | GCGGATCCGATAGCTCTTTGCCATTAAC |
| 9 - *IR4AG* | TTCGCAATTTGCTAcgcagacCCGCCGCTTGTGC |
| 10 - *IR4AD* | GCACAAGCGGCGGgtctgcgTAGCAAATTGCGAA |
| 11 - *IR4BG* | TTGCTAGCGTCTGCaGaCGCTTGTGCTAAAA |
| 12 - *IR4BD* | TTTTAGCACAAGCGtCtGCAGACGCTAGCAA |
| 13 - *IR4CG* | GCAATTTGCTAGCGgCgGCCGCCGCTTGTGC |
| 14 - *IR4CD* | GCACAAGCGGCGGCcGcCGCTAGCAAATTGC |
| 15 - *IR3aG* | CTAGGGCAGGATTCtagAaTTGCTAGCGTCTGC |
| 16 - *IR3aD* | GCAGACGCTAGCAAtTctaGAATCCTGCCCTAG |
| 17 - *mobCT* | GCGAGCTCCTTCATCGATCCCCCACTTG |
| 18 - MOBC155 | CGGTCGACTATTACTTAGTTTAATCGAT |
| *19- oriT65G* | AAACATGGGGGGGTTAGAACTAGGGCAGGATTCGCA  ATTTGCTAGCGTCTGCCGCCGCTTG |
| *20 - oriT65D* | CAAGCGGCGGCAGACGCTAGCAAATTGCGAATCCTGC  CCTAGTTCTAACCCCCCCATGTTT |

^1^The restriction enzyme recognition sites are underlined, start codon is in bold. Substituted nucleotides in primers for PCR mutagenesis are indicated by lower case.
