# Supplementary figures and images for: MobC of conjugative RA3 plasmid from IncU group autoregulates the expression of bicistronic mobC-nic operon and stimulates conjugative transfer
Source: BMC Microbiol. 2014 Sep 4;14:235. doi: 10.1186/s12866-014-0235-1 (PMC4175270; doi:10.1186/s12866-014-0235-1)

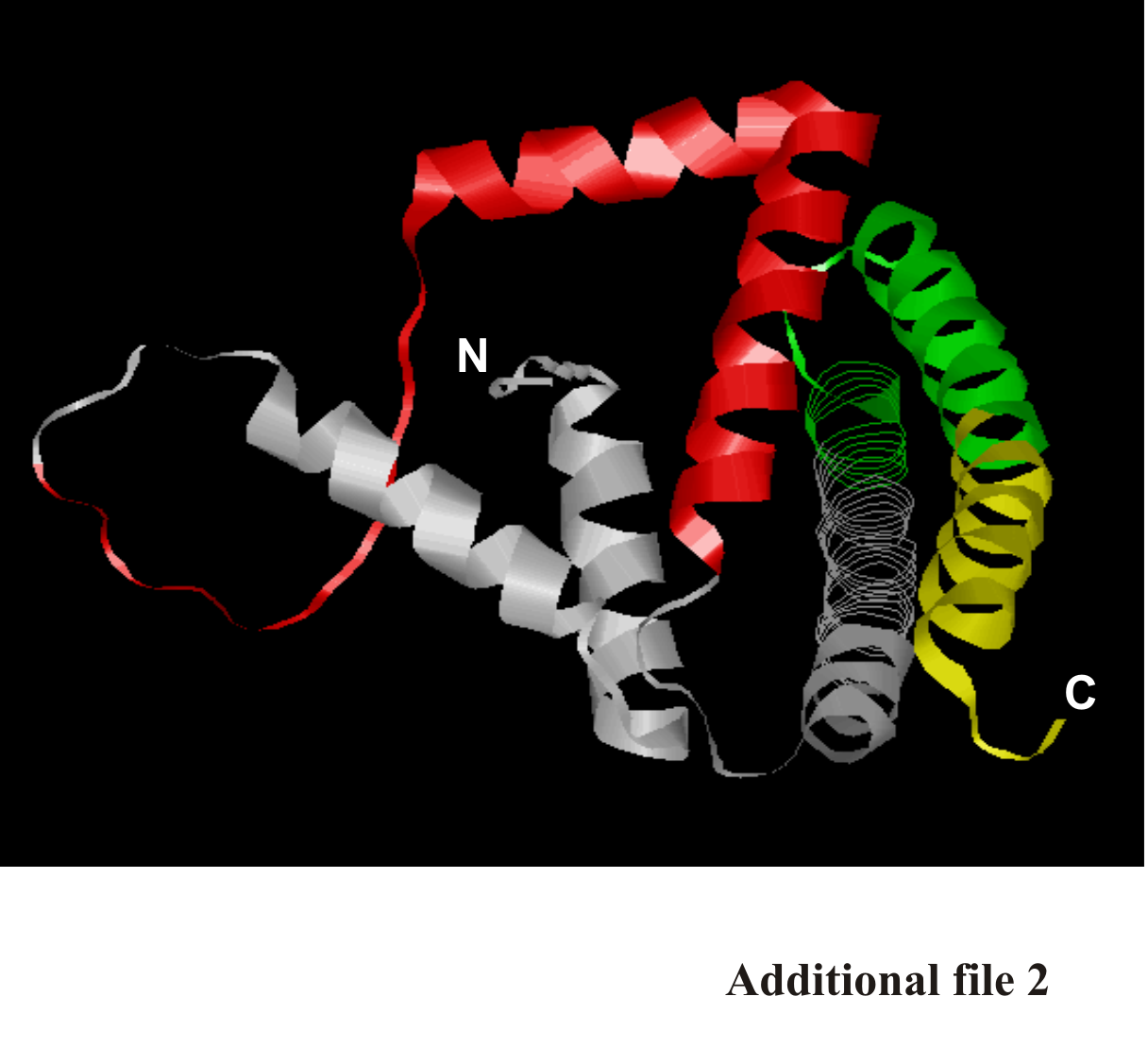

Supplement: Additional file 2: — 3D structure of MobC predicted by I-TASSER online server. The putative RHH motif (52 aa- 99 aa) is marked red, a bacterial mobilization protein motif (117 aa-133 aa) is located mainly in helix 5 and indicated by four thin lines. The region of 19 aa deleted in MobC1-155 is colored yellow, the deletion in MobC1-129 encompasses amino acids marked yellow and green. [file 12866_2014_235_MOESM2_ESM.tiff]

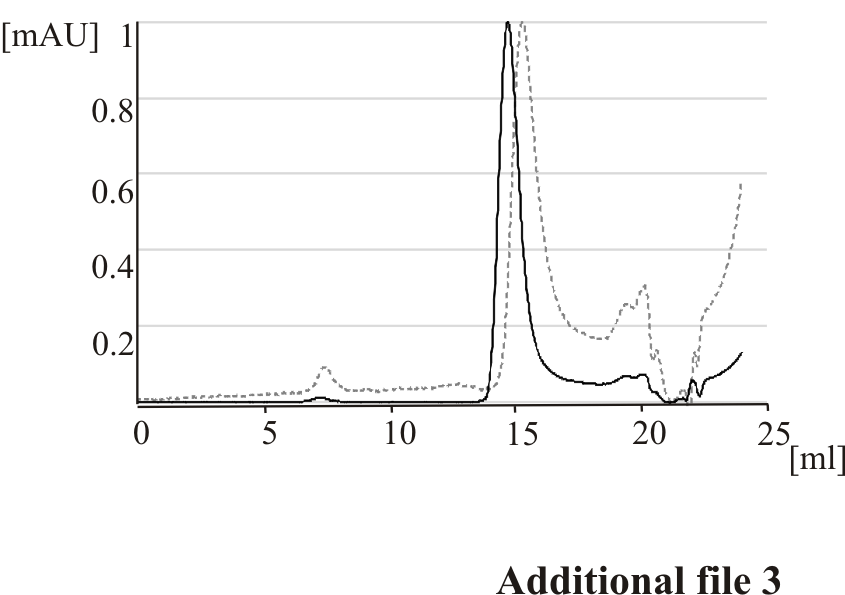

Supplement: Additional file 3: — The gel filtration chromatography of MobC and MobC1-129. The 0.5 ml of purified His6-MobC (0.24 mg ml-1) and His6-MobC1-129 (0.04 mg ml-1) were loaded independently on Superdex 200 10/300 GL column (GE Healthcare) equilibrated with size-exclusion chromatography buffer (10 mM Tris pH 8.0, 150 mM NaCl) with three in-line detectors: UV (ÄKTA Purifier, GE Healthcare), MALS (DAWN HELEOS-II, Wyatt Technology) and differential refractometer (Optilab T-rEX, Wyatt Technology). Data processing and molecular weight calculations were performed using ASTRA software (Wyatt Technology). Diagram presents relative units (mAU) calculated from the absorbance values of UV 280 nm. The black line corresponds to the gel filtration profile of MobC whereas the grey dashed line indicates gel filtration profile of MobC1-129. Molecular weight of complexes His6-MobC and His6-MobC1-129 are 49 kDa and 42 kDa respectively. Data from mass spectrometry show that molecular weight of His6-MobC monomer is 24 kDa and His6-MobC1-129 monomer is 18 kDa (Technical specification: Spectrometer Synapt G2 MS (Waters), LC system nanoACQUITY (Waters), Trap Column ACQUITY UPLC PrST C4 VanGuard, Pre-column 300A, 1.7 μm, 2.1 mm, 5 mm). [file 12866_2014_235_MOESM3_ESM.tiff]

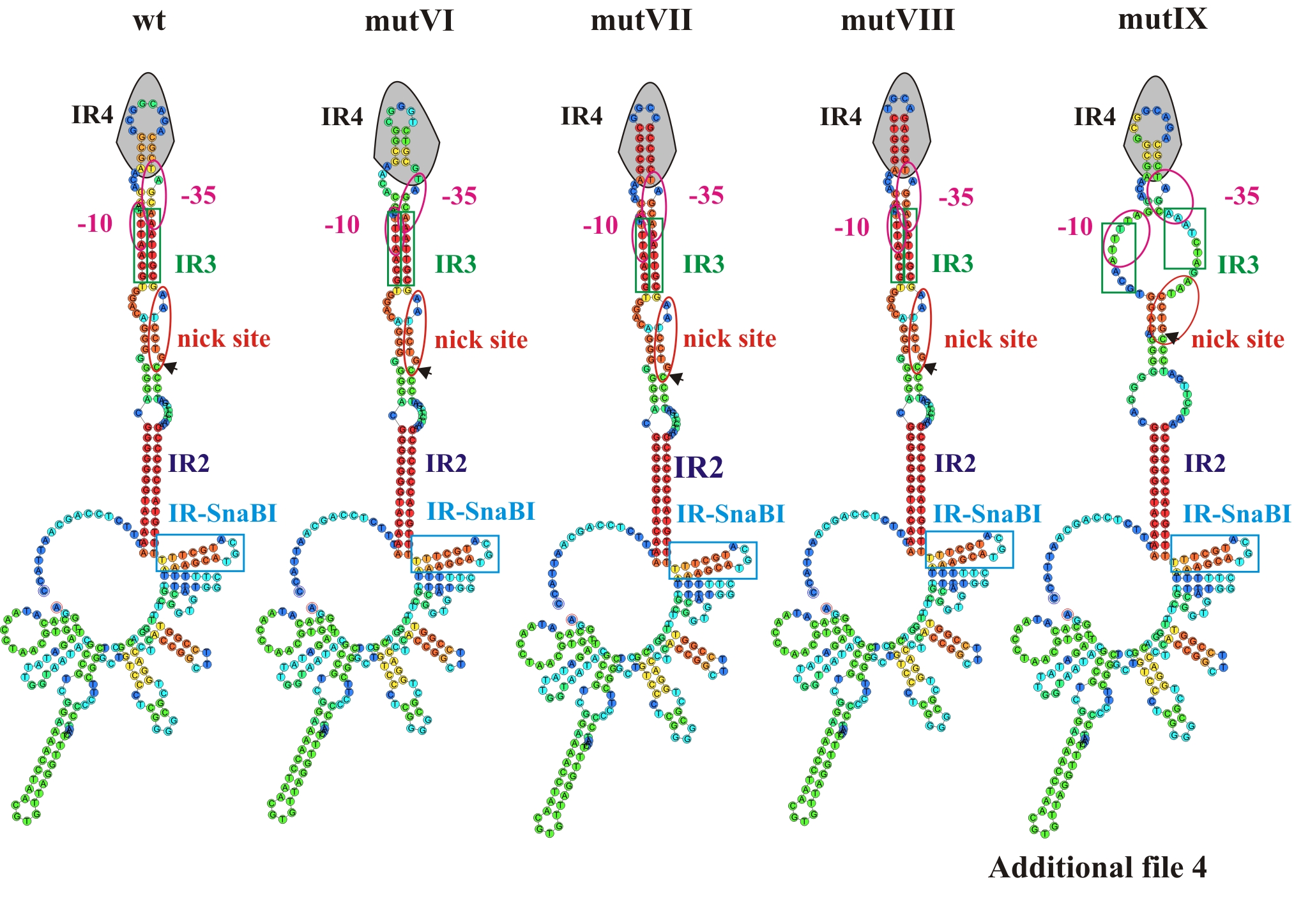

Supplement: Additional file 4: — Single stranded DNA folding of oriT RA3 region and its mutant derivatives. The MobC binding site (IR4) is shadowed in grey, the -35 and -10 sequences of mobCp are circled in pink, the nick site is circled in red with arrows pointing the site of cleavage. The IR3 and IR-SnaBI are marked as green or blue rectangles, respectively. The mutants are designated as on Figure 4A. The DNA sequence analyzed by Geneious6.1.3 encompasses region of RA3 between 9560-9840 nt. [file 12866_2014_235_MOESM4_ESM.jpeg]
